# Supplementary material for: Phosphoproteomics of aged insulin-resistant bone identifies P70S6K phosphorylation of AFF4 as a gene-specific transcriptional regulator
Source: Nat Commun. 2025 Dec 31;17:1347. doi: 10.1038/s41467-025-68106-4 (PMC12873371; doi:10.1038/s41467-025-68106-4)
Supplement: Supplementary file 1 — Supplementary Information [file 41467_2025_68106_MOESM1_ESM.pdf]

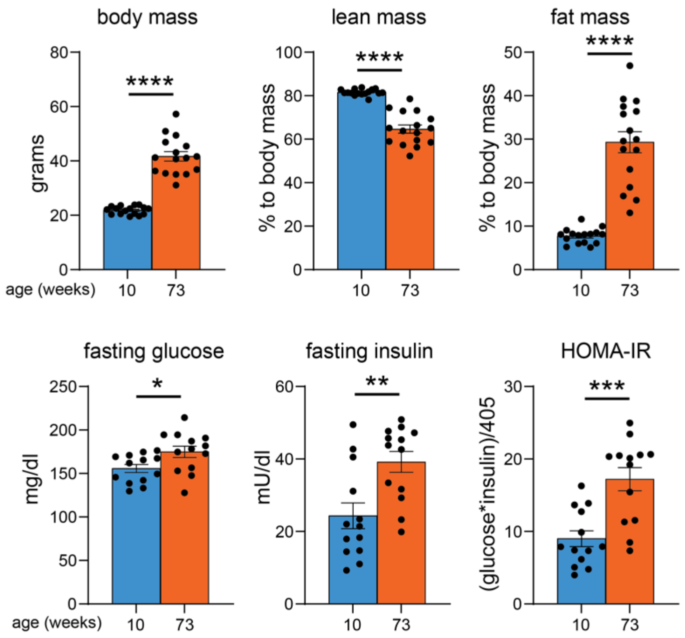

Supplementary Figure 1. Metabolic phenotyping of age stratified cohort. Body composition, fasting blood glucose, insulin and Homeostatic Model Assessment for Insulin Resistance (HOMA-IR) of 10-week and 73-week-old mice. *p-value<0.05; **p-value<0.01, ***p-value<0.005, ****p-value<0.001; unpaired t-test. Source data are provided as a Source Data file.

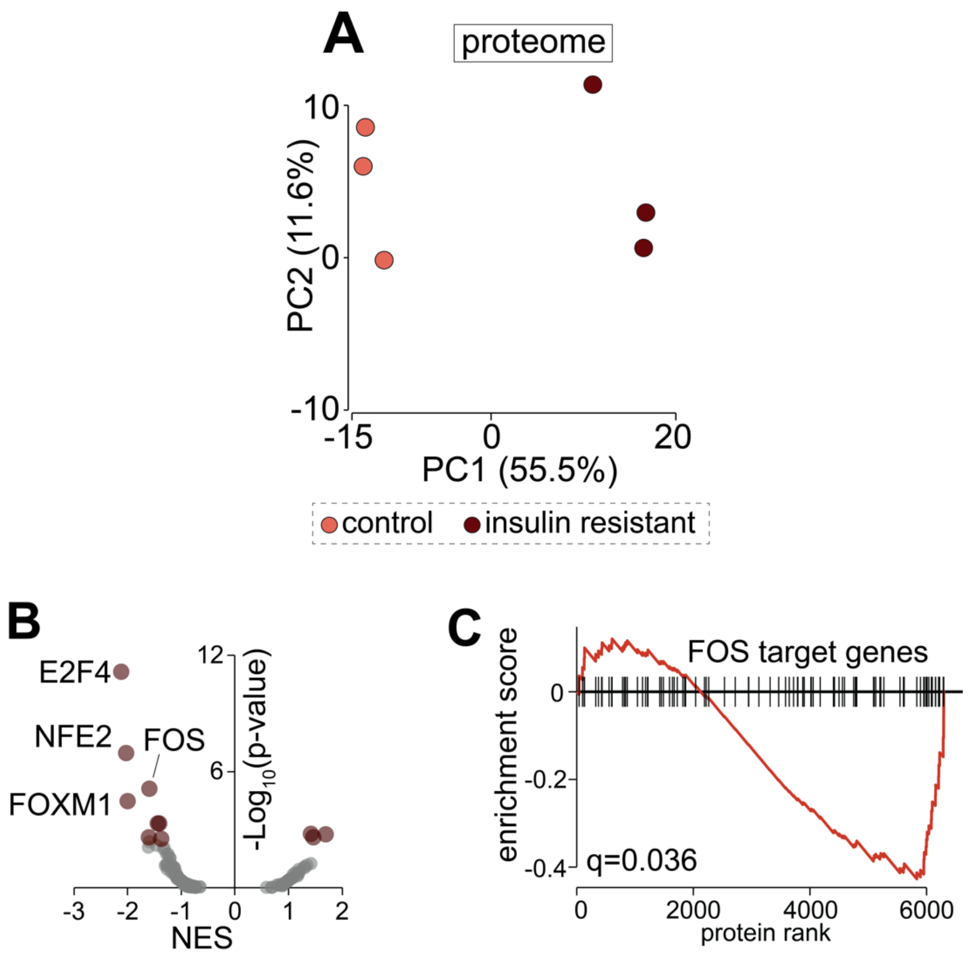

Supplementary Figure 2. Proteomic analysis of control vs hyperinsulinemia-induced insulin-resistant Kusa 4B10 osteoblasts . (**A**) Principal component analysis (PCA) of the proteomic data. (**B**) Proteomic GSEA of transcriptional regulators from the ChEA3 database, and (**C**) individual plot of FOS target genes.

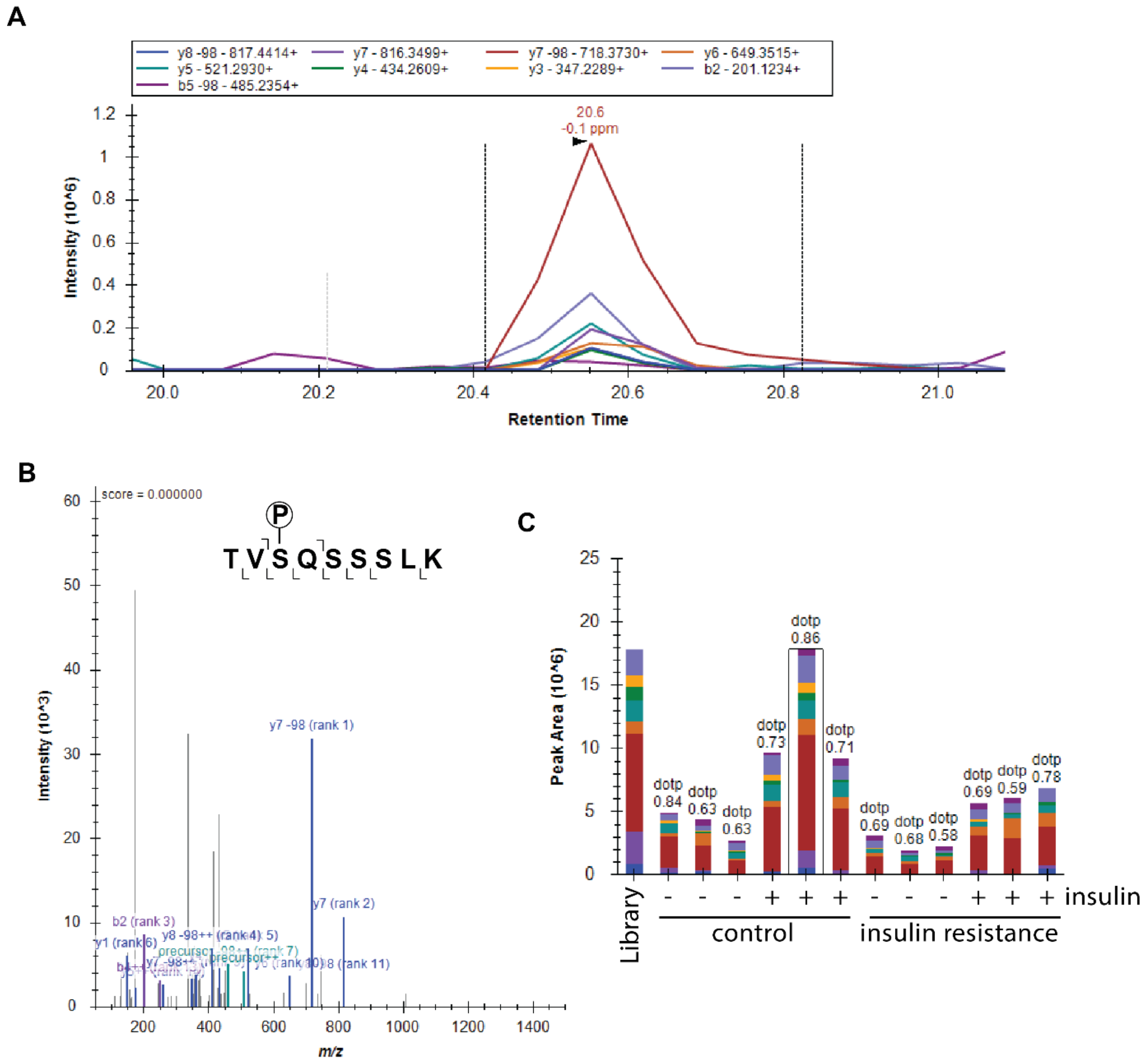

Supplementary Figure 3. Targeted phosphoproteomics of S831 phosphorylation on AFF4 in insulin-resistant osteoblasts with or without 20 min of insulin stimulation. Screen shot from Skyline targeted analysis. **(A)** Example of MS/MS extracted ion chromatogram. **(B)** MS/MS spectral library generated using data-dependent acquisition on immunoprecipitated FLAG-AFF4-WT from transgenic HEK293T cells. **(C)** Extracted ion chromatogram area under the curve. Source data for Figure 5D.

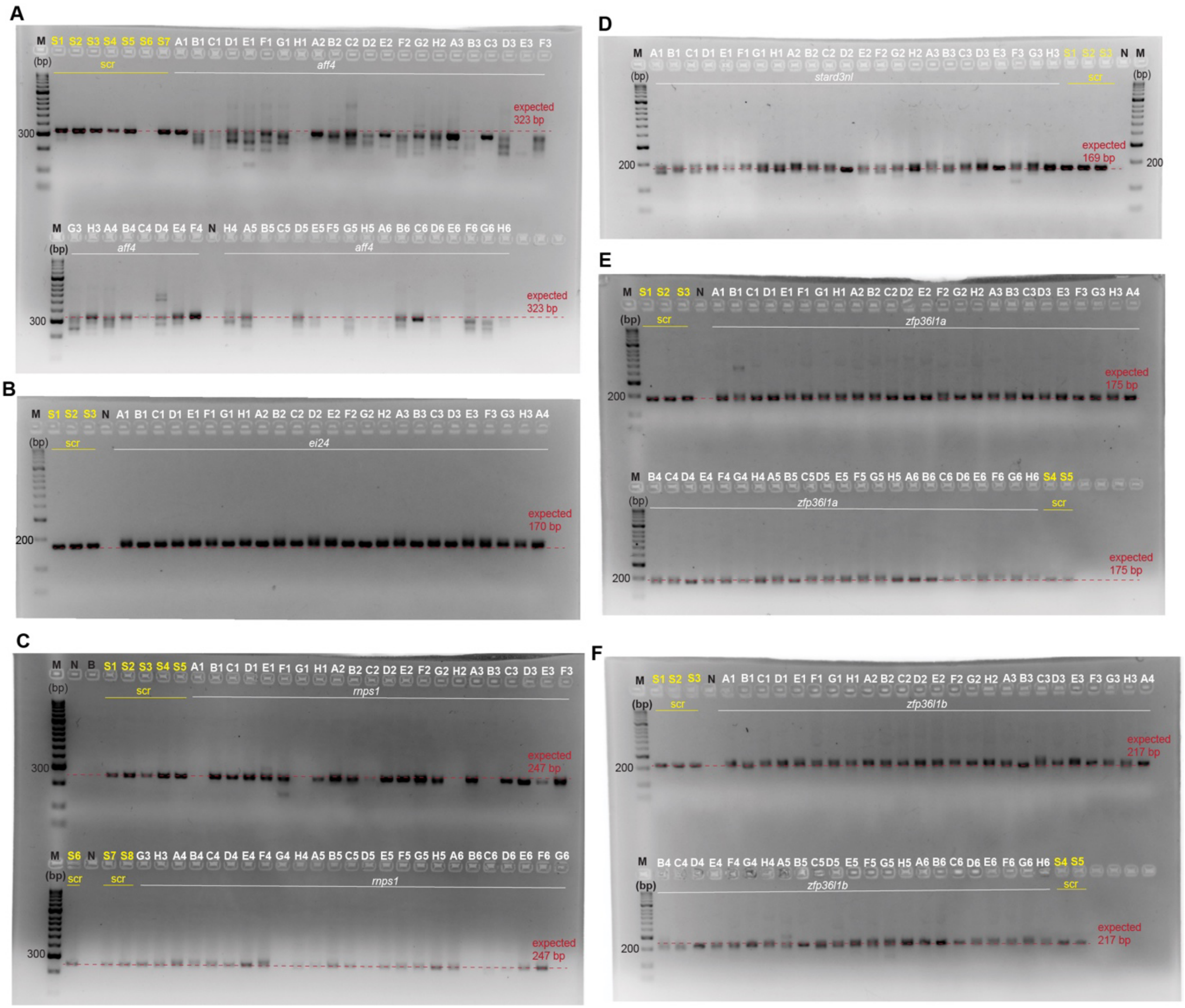

Supplementary Figure 4. PCR genotyping of F0 crispants positive hits in the zebrafish screen (A) *aff4* F0 crispants, **(B)** *ei24* F0 crispants, **(C)** *rnps1* F0 crispants, **(D)** *stard3nl* F0 crispants, **(E)** *zfp36l1a* F0 crispants, **(F)** *zfp36l1b* F0 crispants

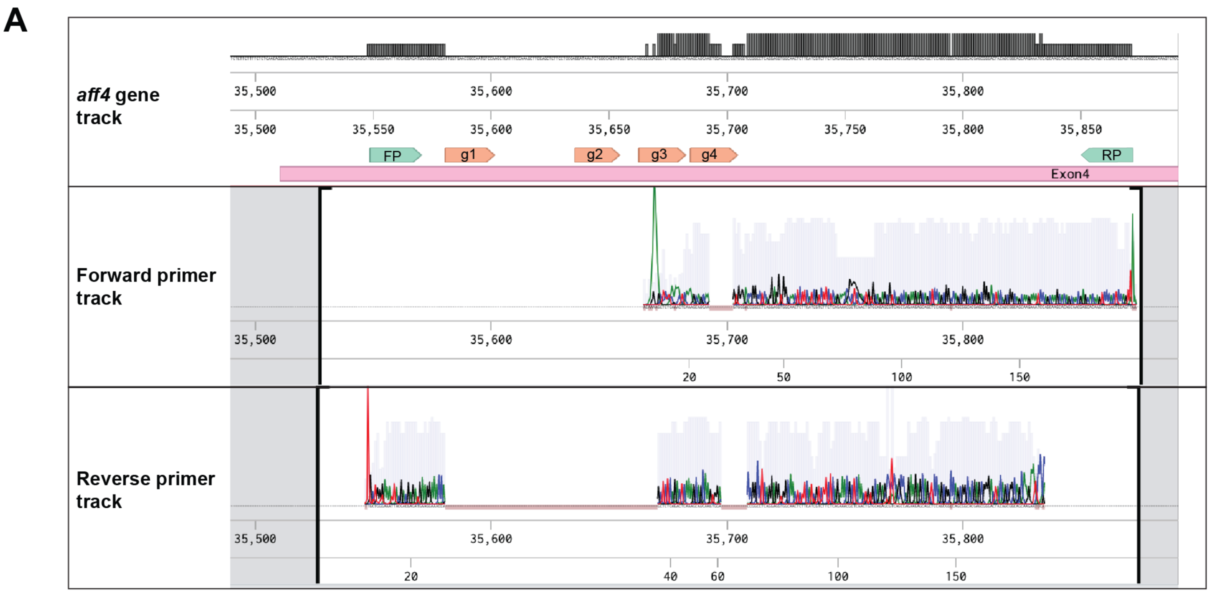

B

```
                35507                                                                           35588
aff4-WT    ...  ACAGGCCAAGGAAGATAAACTCTCAAGTCGCATCCAGAGCATGCTGGGAAATTACGACGAGATGAAGGAAACCATTGGTGAA
aff4_F1_R...    ----------------------------------------TTGCTGGGAAATTACGACGAGATGAAGGAAACCA--------
aff4_F1_F...    ----------------------------------------------------------------------------------

                35589                                                                           35670
aff4-WT    ...  CCGCCAATGTCCAAGCTCATTTCCAAAGCTTCCAGCTCTTCCTCCGAGGATAAATCTGGCCAGTATGGTGACCAGCGCGGAG
aff4_F1_R...    ----------------------------------------------------------------------------------
aff4_F1_F...    ----------------------------------------------------------------------------ACAAAA

                35671                                                                           35752
aff4-WT    ...  GCTCTCAGACTCAAAGCAGCAAGTGGACCCCGGTGGGTCCGGCCTCAGGAGGTGGCAACTCTTCATCGTCTTCTCAGAAACG
aff4_F1_R...    GCTCTCAGACTCAAAGCAGCAAGTGGA-----------CCGGCCTCAGGAGGTGGCAACTCTTCATCGTCTTCTCAGAAACG
aff4_F1_F...    GCTCTCAAACTCAAAGCAGCAA----------GTGGGACCGGCCTCAGGAGGTGGCAACTCTTCATCGTCTTCTCAGAAACG

                35753                                                                           35834
aff4-WT    ...  CTCAACTGTGCAGAGCGTCAGCCAGAAGAGCAGCTCCAGCGGGCAGCGGCACGAGCGGGACTACAGCGGCAGCAAGAAATCC
aff4_F1_R...    CTCAACTGTGCAGAGCGTCAGCCAGAAGAGCAGCTCCAGCGGCCAGCGGCACGAGCGGGACTACAGCGGCAGCAAGAATCCA
aff4_F1_F...    CTCAACTGTGCAGAGCGTCAGCCAGAAGAGCAGCTCCAGCGGCCAGCGGCACGAGCGGGACTACAGCGGCAGCAAGAAATCC

                35835                                                                           35916
aff4-WT    ...  AGCAAGCACAGCAACGAGCACAAGTCCCACTCCAGTTCCAGCCCGGCCAAAGTCTCCAGCAACCACTCGCGCCGAGTCCCCA
aff4_F1_R...    ----------------------------------------------------------------------------------
aff4_F1_F...    AGCAAGCACAGCAACGAGCACAAGTCCCACTCCAGTT---------------------------------------------
```

Supplementary Figure 5: *aff4* germline mutant Sanger sequencing. Sanger sequencing of the *aff4* F1 germline mutant with the **(A)** sequencing track and **(B)** multiple sequence alignment showing the region of germline editing (in orange). FP= forward primer, RP= reverse primer, g1= sgRNA1, g2=sgRNA2, g3=sgRNA3, g4=sgRNA4, *aff4*-WT= wild type gene sequence, *aff4*_F1_R= reverse primer sequenced region, *aff4*_F1_F= forward primer sequenced region.

Supplementary Table 1: Key resource table

RESOURCE	SOURCE	IDENTIFIER
**Experimental Models: Cell Lines**		
HEK-293T	ATCC	CRL-1573
Kusa 4B10	PMID: 12938165	
**Experimental Models: Mice**		
C57BL/6J	Animal Resource Centre (Australia)	JAX 000664
**Experimental Models: Zebrafish**		
AB Wildtype	The Zebrafish Information Network	ZDB-GENO-960809-7
**Antibodies**		
Anti-Acetyl-Histone-H4	Millipore	06-866 (RRID: AB_310270)
Anti-Phospho-Akt (Ser473) (D9E)	Cell Signaling Technology	4060 (RRID: AB_2315049)
Anti-Akt	Cell Signaling Technology	9272 (RRID: AB_329827)
Anti-Phospho-Akt Substrate (RXRXXS/T) (110B7E)	Cell Signaling Technologies	9614 (RRID: AB_331810)
Anti-FLAG-M2	Sigma	F1804 (RRID: AB_262044)
Ultra-LEAF non-specific negative control IgG	BioLegend	401507 (RRID: AB_2893160)
Anti-MLLT1/ENL(D9M4B)	Cell Signaling Technology	14893 (RRID: AB_2798636)
Anti-Phospho-p70 S6 Kinase (Thr389) (108D2)	Cell Signaling Technologies	9234 (RRID: AB_2269803)
Anti-p70 S6 Kinase	Cell Signaling Technologies	9202 (RRID: AB_331676)
Anti-Phospho-PRAS40 (Thr246) (C77D7)	Cell Signaling Technologies	2997 (RRID: AB_2258110)
Anti-PRAS40 (D23C7)	Cell Signaling Technologies	2691 (RRID: AB_2225033)
Anti-RNA polymerase II subunit B1 (phospho CTD Ser-2)	Millipore	04-1571 (RRID: AB_11212363)
Anti-RNA pol II CTD Phospho Ser5	Active Motif	61085 (RRID: AB_2687451)
Anti-RNA polymerase II (8WG16)	Santa Cruz Biotechnology	sc-56767 (RRID: AB_785522)
Anti-Phospho-S6 Ribosomal Protein (Ser235/236)	Cell Signaling Technologies	2211 (RRID: AB_331679)
Anti-S6 Ribosomal Protein (54D2)	Cell Signaling Technologies	2317 (RRID: AB_2238583)
Anti-Histone H3 (D1H2)	Cell Signaling Technologies	4499 (RRID:AB_10544537)
Anti- Crotonyl-Histone H3 (Lys18) (E8D9M)	Cell Signaling Technologies	69465 (RRID:AB_3676457)
**Deposited Data**		
Phosphoproteomic and proteomics of insulin	This paper	PRIDE: PXD054205

signaling in aged mouse bone		Project Webpage:https://www.ebi.ac.uk/pride/archive/projects/PXD054205<br>FTP Download:https://ftp.pride.ebi.ac.uk/pride/data/archive/2025/12/PXD054205
Zebrafish caudal fin phosphoproteomic of Rps6kb1a/b knockdown	This paper	PRIDE: PXD054212<br>Project Webpage:https://www.ebi.ac.uk/pride/archive/projects/PXD054212<br>FTP Download:https://ftp.pride.ebi.ac.uk/pride/data/archive/2025/12/PXD054212
Analysis of insulin-regulated phosphorylation of AFF4 with Akti or S6Ki and S6K in vitro kinase assay	This paper	PRIDE: PXD054247<br>Project Webpage:https://www.ebi.ac.uk/pride/archive/projects/PXD054247<br>FTP Download:https://ftp.pride.ebi.ac.uk/pride/data/archive/2025/12/PXD054247
Affinity purification – mass spectrometry of AFF4 WT or S829/S831/3/5/8A mutant	This paper	PRIDE: PXD054250<br>Project Webpage:https://www.ebi.ac.uk/pride/archive/projects/PXD054250<br>FTP Download:https://ftp.pride.ebi.ac.uk/pride/data/archive/2025/12/PXD054250
Targeted phosphoproteomics of mouse S831 AFF4 phosphorylation in control or insulin resistant osteoblasts	This paper	Panorama Web: U of Melbourne – Parker Lab: PRM of mouse AFF4 S831 phosphorylation
Proteomic and secretomic analysis of Kusa 4B10 osteoblasts	This paper	PRIDE: PXD054479<br>Project Website:https://www.ebi.ac.uk/pride/archive/projects/PXD054479<br>FTP Download:https://ftp.pride.ebi.ac.uk/pride/data/archive/2025/12/PXD054479
Transcriptomics of HEK293T cells expressing AFF4-wild type or AFF4-T829/S831/S833/S834/S835A mutant treated with or without insulin	This paper	NCBI – SRA: PRJNA1146056 Temporary Submission ID: SUB14629593<br>https://www.ncbi.nlm.nih.gov/sra
Source data	This paper	Unprocessed source data underlying all blots and graphs. Related to Figures 1, 2, 3,4, 5, and 6. The file also includes sequencing results to confirm *aff4* CRISPR edits.
**Software and Algorithms**		
R version 4.1.1	R Development Core Team, 2016	https://www.R-project.org/
MaxQuant 1.6.7.0 & 1.6.12.0	PMID: 19029910	http://www.biochem.mpg.de/5111795/maxquant
Perseus Plugin Peptide Collapse	PMID: 32034161	https://github.com/AlexHgO/Perseus_Plugin_Peptide_Collapse

KSP-PUEL	PMID: 26395771	https://github.com/PYangLab/KSP-PUEL
NormalyzerDE	PMID: 30277078	https://www.bioconductor.org/packages/release/bioc/html/NormalyzerDE.html
ImageJ 1.53j		http://imagej.nih.giv/ij
ImageJ ZFBONE plugin	PMID: 32534223	https://github.com/MarcoTarasco/ZFBONE
ImageJ Skeleton length plugin	PMID: 15822812	https://dev.mri.cnrs.fr/projects/imagej-macros/wiki/Measure_Skeleton_Length
ImageLab 4.1	Chemidoc-MP Imaging System	Bio-Rad, Australia
Leica Application Suite X, version 4.13.0	Leica, Germany	
GraphPad Prism 10.0	GraphPad	
