# Supplementary material for: Phosphoproteomics of aged insulin-resistant bone identifies P70S6K phosphorylation of AFF4 as a gene-specific transcriptional regulator
Source: Nat Commun. 2025 Dec 31;17:1347. doi: 10.1038/s41467-025-68106-4 (PMC12873371; doi:10.1038/s41467-025-68106-4)
Supplement: Supplementary file 2 — Description of Additional Supplementary Files [file 41467_2025_68106_MOESM2_ESM.pdf]

Description of Additional Supplementary Files

File Name: Supplementary Data 1
Description: Phosphoproteomic analysis of 10-week versus 73-week mouse tibiae tissue in the presence and absence of insulin stimulation. Full dataset including two-sided limma moderated t-test with Benjamini Hochberg FDR reporting the calculated p-value and q-value for each phosphosite.

File Name: Supplementary Data 2
Description: Proteomic analysis of 10-week versus 73-week mouse tibiae tissue. Full dataset including the limma moderated t-test with Benjamini Hochberg FDR reporting the calculated p-value and q-value for each protein.

File Name: Supplementary Data 3
Description: Phosphoproteomic dataset of wild-type zebrafish caudal fin clippings.

File Name: Supplementary Data 4
Description: CRISPR/Cas9 functional genomic screen in zebrafish measuring body length, bone mineralization and skeleton length. Full dataset including unpaired, two-sided t-test reporting the calculated p-value for each measurement.

File Name: Supplementary Data 5
Description: *aff4* and scr crispant size bins and spine development metrics

File Name: Supplementary Data 6
Description: Proteomic analysis of *aff4* zebrafish caudal fin. Full dataset including two-sided unpaired t-test with Benjamini Hochberg FDR reporting the calculated p-value and q-value for each protein.

File Name: Supplementary Data 7
Description: Phosphoproteomic analysis of AFF4. Full dataset quantifying 47 phosphosites with unpaired, two-sided t-test reporting the calculated p-value for each phosphosite.

File Name: Supplementary Data 8
Description: Phosphoproteomic analysis of *rps6kb1/2* zebrafish caudal fins. Full dataset including two-sided unpaired t-test with permutation-based FDR reporting the calculated p-value.

File Name: Supplementary Data 9
Description: Proteomic analysis of insulin-resistant Kusa 4B10 osteoblasts. Full dataset including two-sided unpaired t-test with permutation-based FDR reporting the calculated p-value and q-value for each protein.

File Name: Supplementary Data 10
Description: Total RNAP2 ChIP-seq analysis of insulin-resistant Kusa 4B10 osteoblasts. Full dataset including two-sided unpaired t-test reporting the calculated p-value.

File Name: Supplementary Data 11
Description: Transcriptomic analysis of HEK293 cells expressing FLAG-tagged AFF4-WT and AFF4-A. Full dataset including two-sided unpaired t-test with Benjamini Hochberg FDR reporting p-value and q-value for each gene.

File Name: Supplementary Data 12
Description: AFF4 interactome using affinity-purification mass spectrometry. Full dataset including two-sided unpaired t-test with permutation-based FDR reporting p-value and q-value for each protein.

File Name: Supplementary Data 13
Description: Complete single guide RNA (sgRNA), constant oligonucleotide and genotyping oligonucleotide sequence information used in the CRISPR/Cas9 zebrafish functional genomic screen.
