# Supplementary material for: Phosphoproteomics of aged insulin-resistant bone identifies P70S6K phosphorylation of AFF4 as a gene-specific transcriptional regulator
Source: Nat Commun. 2025 Dec 31;17:1347. doi: 10.1038/s41467-025-68106-4 (PMC12873371; doi:10.1038/s41467-025-68106-4)
Supplement: Supplementary file 4 — Reporting Summary [file 41467_2025_68106_MOESM4_ESM.pdf]

nature portfolio

Corresponding author(s): Benjamin L. Parker

Last updated by author(s): Dec 3, 2025

Reporting Summary

Nature Portfolio wishes to improve the reproducibility of the work that we publish. This form provides structure for consistency and transparency in reporting. For further information on Nature Portfolio policies, see our Editorial Policies and the Editorial Policy Checklist.

Statistics

For all statistical analyses, confirm that the following items are present in the figure legend, table legend, main text, or Methods section.

n/a	Confirmed	
☐	☒	The exact sample size (*n*) for each experimental group/condition, given as a discrete number and unit of measurement
☐	☒	A statement on whether measurements were taken from distinct samples or whether the same sample was measured repeatedly
☐	☒	The statistical test(s) used AND whether they are one- or two-sided<br>*Only common tests should be described solely by name; describe more complex techniques in the Methods section.*
☒	☐	A description of all covariates tested
☐	☒	A description of any assumptions or corrections, such as tests of normality and adjustment for multiple comparisons
☒	☐	A full description of the statistical parameters including central tendency (e.g. means) or other basic estimates (e.g. regression coefficient) AND variation (e.g. standard deviation) or associated estimates of uncertainty (e.g. confidence intervals)
☐	☒	For null hypothesis testing, the test statistic (e.g. *F*, *t*, *r*) with confidence intervals, effect sizes, degrees of freedom and *P* value noted<br>*Give P values as exact values whenever suitable.*
☒	☐	For Bayesian analysis, information on the choice of priors and Markov chain Monte Carlo settings
☒	☐	For hierarchical and complex designs, identification of the appropriate level for tests and full reporting of outcomes
☒	☐	Estimates of effect sizes (e.g. Cohen's *d*, Pearson's *r*), indicating how they were calculated

Our web collection on statistics for biologists contains articles on many of the points above.

Software and code

Policy information about availability of computer code

Data collection	All information has been provided in the methods section.

Data analysis	Details of all data analysis has been provided in the Methods. Details of software used is provided as follows:<br>R version 4.1.1 R Development Core Team, 2016 https://www.R-project.org/<br>MaxQuant 1.6.7.0 & 1.6.12.0 PMID: 19029910 http://www.biochem.mpg.de/5111795/maxquant<br>Perseus Plugin Peptide Collapse PMID: 32034161 https://github.com/AlexHgO/Perseus_Plugin_Peptide_Collapse<br>KSP-PUEL PMID: 26395771<br>https://github.com/PYangLab/KSP-PUEL<br>NormalyzerDE PMID: 30277078<br>https://www.bioconductor.org/packages/release/bioc/html/NormalyzerDE.html<br>ImageJ 1.53j http://imagej.nih.giv/ij<br>ImageJ ZFBONE plugin PMID: 32534223 https://github.com/MarcoTarasco/ZFBONE<br><br>ImageJ Skeleton length plugin PMID: 15822812 https://dev.mri.cnrs.fr/projects/imagej-macros/wiki/Measure_Skeleton_Length<br>ImageLab 4.1 Chemidoc-MP Imaging System<br>Bio-Rad, Australia<br>Leica Application Suite X, version 4.13.0 Leica, Germany<br>GraphPad Prism 10.0 GraphPad

For manuscripts utilizing custom algorithms or software that are central to the research but not yet described in published literature, software must be made available to editors and reviewers. We strongly encourage code deposition in a community repository (e.g. GitHub). See the Nature Portfolio guidelines for submitting code & software for further information.

Data

Policy information about availability of data

All manuscripts must include a data availability statement. This statement should provide the following information, where applicable:

- Accession codes, unique identifiers, or web links for publicly available datasets
- A description of any restrictions on data availability
- For clinical datasets or third party data, please ensure that the statement adheres to our policy

Phosphoproteomic and proteomics of insulin signaling in aged mouse bone This paper PRIDE: PXD054205
Username: reviewer_pxd054205@ebi.ac.uk
Password: 8ZPOAq6TitF2
Zebrafish caudal fin phosphoproteomic of Rps6kb1a/b knockdown This paper PRIDE: PXD054212
Username: reviewer_pxd054212@ebi.ac.uk
Password: lLdENPAjFkJx
Analysis of insulin-regulated phosphorylation of AFF4 with Akti or S6Ki and S6K in vitro kinase assay This paper PRIDE: PXD054247
Username: reviewer_pxd054247@ebi.ac.uk
Password: kBaKiZpuAD4P
Affinity purification – mass spectrometry of AFF4 WT or S829/S831/3/5/8A mutant This paper PRIDE: PXD054250
Username: reviewer_pxd054250@ebi.ac.uk
Password: 1nu2Wkjl8MlU
Targeted phosphoproteomics of mouse S831 AFF4 phosphorylation in control or insulin resistant osteoblasts This paper Panorama Web: U of Melbourne – Parker Lab: PRM of mouse AFF4 S831 phosphorylation
Proteomic and secretomic analysis of Kusa 4B10 osteoblasts This paper PRIDE: PXD054479
Username: reviewer_pxd054479@ebi.ac.uk
Password: 0gHY5D1easqC
Transcriptomics of HEK293T cells expressing AFF4-wild type or AFF4-T829/S831/S833/S834/S835A mutant treated with or without insulin This paper
NCBI – SRA: PRJNA1146056 Temporary Submission ID: SUB14629593
https://www.ncbi.nlm.nih.gov/sra

Research involving human participants, their data, or biological material

Policy information about studies with human participants or human data. See also policy information about sex, gender (identity/presentation), and sexual orientation and race, ethnicity and racism.

Reporting on sex and gender	No human data are presented
Reporting on race, ethnicity, or other socially relevant groupings	No human data are presented
Population characteristics	No human data are presented
Recruitment	No human data are presented
Ethics oversight	No human data are presented

Note that full information on the approval of the study protocol must also be provided in the manuscript.

Field-specific reporting

Please select the one below that is the best fit for your research. If you are not sure, read the appropriate sections before making your selection.

- [x] Life sciences
- [ ] Behavioural & social sciences
- [ ] Ecological, evolutionary & environmental sciences

For a reference copy of the document with all sections, see nature.com/documents/nr-reporting-summary-flat.pdf

Life sciences study design

All studies must disclose on these points even when the disclosure is negative.

Sample size	Sample size calculations were not performed but based on previous literature:<br>Mouse experiments n=8-16<br>zebrafish experiments n=30-100<br>cell culture experiments n=2-3 (genomic/transcriptomics experiments), n=3-4 (proteomics experiments), n=3-4 (signaling, cell biology and biochemistry experiments)
Data exclusions	no data were excluded
Replication	Biological replicates are indicated throughout the manuscript.
Randomization	Young or old mice were randomly allocated to vehicle control or acute insulin stimulation. Zebrafish embryos were randomly assigned to either scramble control gRNA or target gRNA injections.
Blinding	no blinding was performed.

Reporting for specific materials, systems and methods

We require information from authors about some types of materials, experimental systems and methods used in many studies. Here, indicate whether each material, system or method listed is relevant to your study. If you are not sure if a list item applies to your research, read the appropriate section before selecting a response.

Materials & experimental systems

n/a	Involved in the study	
[ ]	[x]	Antibodies
[ ]	[x]	Eukaryotic cell lines
[x]	[ ]	Palaeontology and archaeology
[ ]	[x]	Animals and other organisms
[x]	[ ]	Clinical data
[x]	[ ]	Dual use research of concern
[x]	[ ]	Plants

Methods

n/a	Involved in the study	
[ ]	[x]	ChIP-seq
[x]	[ ]	Flow cytometry
[x]	[ ]	MRI-based neuroimaging

Antibodies

Antibodies used

Anti-Acetyl-Histone-H4 Millipore 06-866 (RRID: AB_310270)
Anti-Phospho-Akt (Ser473) (D9E) Cell Signaling Technology 4060 (RRID: AB_2315049)
Anti-Akt Cell Signaling Technology 9272 (RRID: AB_329827)
Anti-Phospho-Akt Substrate (RXRXXS/T) (110B7E) Cell Signaling Technology 9614 (RRID: AB_331810)
Anti-FLAG-M2 Sigma F1804 (RRID: AB_262044)
Ultra-LEAF non-specific negative control IgG BioLegend 401507 (RRID: AB_2893160)
Anti-MLLT1/ENL(D9M4B) Cell Signaling Technology 14893 (RRID: AB_2798636)
Anti-Phospho-p70 S6 Kinase (Thr389) (108D2) Cell Signaling Technology 9234 (RRID: AB_2269803)
Anti-p70 S6 Kinase Cell Signaling Technology 9202 (RRID: AB_331676)
Anti-Phospho-PRAS40 (Thr246) (C77D7) Cell Signaling Technology 2997 (RRID: AB_2258110)
Anti-PRAS40 (D23C7) Cell Signaling Technology2691 (RRID: AB_2225033)
Anti-RNA polymerase II subunit B1 (phospho CTD Ser-2) Millipore 04-1571 (RRID: AB_11212363)
Anti-RNA pol II CTD Phospho Ser5 Active Motif 61085 (RRID: AB_2687451)
Anti-RNA polymerase II (8WG16) Santa Cruz Biotechnology sc-56767 (RRID: AB_785522)
Anti-Phospho-S6 Ribosomal Protein (Ser235/236) Cell Signaling Technology 2211 (RRID: AB_331679)
Anti-S6 Ribosomal Protein (54D2) Cell Signaling Technology 2317 (RRID: AB_2238583)
Anti-Histone H3 (D1H2) Cell Signaling Technology 4499 (RRID:AB_10544537)
Anti- Crotonyl-Histone H3 (Lys18) (E8D9M) Cell Signaling Technology 69465 (RRID:AB_3676457)

Validation	We prioritized antibodies from Cell Signaling Technology which all show validation in their data sheets. All other antibodies have been used in previous publications and all show validation in data sheets.

Eukaryotic cell lines

Policy information about cell lines and Sex and Gender in Research

Cell line source(s)	HEK-293T ATCC CRL-1573<br>Kusa 4B10 PMID: 12938165
Authentication	None of the cell lines were authenticated.
Mycoplasma contamination	All cell lines were tested and confirmed free of mycoplasma
Commonly misidentified lines (See ICLAC register)	not applicable.

Animals and other research organisms

Policy information about studies involving animals; ARRIVE guidelines recommended for reporting animal research, and Sex and Gender in Research

Laboratory animals	C57BL/6J Animal Resource Centre (Australia) JAX 000664
Wild animals	Not applicable
Reporting on sex	The sex of animals have been indicated.
Field-collected samples	not applicable
Ethics oversight	All mouse experiments were approved by The University of Melbourne Animal Ethics Committee (AEC ID1914940) and conformed to the Australian code for the care and use of animals for scientific purposes as stipulated by the National Health and Medical Research Council of Australia. C57BL/6J mice (JAX 000664) were obtained from Animal Resource Centre (WA, Australia). Mice were housed at 22°C (+/-1°C) in groups of five/cage and maintained on a standard chow diet (Specialty Feeds, Australia) with a 12 h light/dark cycle and ad libitum access to food and water.

Note that full information on the approval of the study protocol must also be provided in the manuscript.

Plants

Seed stocks	na
Novel plant genotypes	na
Authentication	NA

ChIP-seq

Data deposition

- [x] Confirm that both raw and final processed data have been deposited in a public database such as GEO.
- [x] Confirm that you have deposited or provided access to graph files (e.g. BED files) for the called peaks.

Data access links<br>*May remain private before publication.*	NCBI – SRA: PRJNA1146056 Temporary Submission ID: SUB14629593<br>https://www.ncbi.nlm.nih.gov/sra
Files in database submission	826c8d8e2822680a1cfb4c16b6e8cdc0 *1_HNJ2JDRXY_CGACGTTA-CTCTGGAT_L001_R1.fastq.gz<br>7473c46ddc195b7abf9209867fab3d67 *1_HNJ2JDRXY_CGACGTTA-CTCTGGAT_L001_R2.fastq.gz<br>6ebc91f937616282530a2781c34ba8be *2_HNJ2JDRXY_TACGCCTT-GCTACTCT_L001_R1.fastq.gz<br>36941138281206dd5d3e58a4716449c7 *2_HNJ2JDRXY_TACGCCTT-GCTACTCT_L001_R2.fastq.gz

f8e561358d10afed8172d7f38760673f *3_HNJ2JDRXY_CCGTAAGA-AGAGTCCA_L001_R1.fastq.gz
17e6dafa254af44e3f619be0e3b948cb *3_HNJ2JDRXY_CCGTAAGA-AGAGTCCA_L001_R2.fastq.gz
b9bb1157b3e2328aefee453dfd509bd0 *4_HNJ2JDRXY_ATCACACG-GTAGCGTA_L001_R1.fastq.gz
b43c29568e785f559dc4f2b88414ea4e *4_HNJ2JDRXY_ATCACACG-GTAGCGTA_L001_R2.fastq.gz
332e8a775aa6fd4dceb572f854d80262 *5_HNJ2JDRXY_CACCTGTT-AGGATAGC_L001_R1.fastq.gz
d570ccf5ff8a4628562e2f3938eb6c00 *5_HNJ2JDRXY_CACCTGTT-AGGATAGC_L001_R2.fastq.gz
c63932a9151ab6741c7d4ab987e5595e *6_HNJ2JDRXY_CTTCGACT-GATCTTGC_L001_R1.fastq.gz
788770b215e6bde7e45b14c4144106f7 *6_HNJ2JDRXY_CTTCGACT-GATCTTGC_L001_R2.fastq.gz
3843ebbba345b5678fe43efe702ff6be *7_HNJ2JDRXY_GTTCTCGT-TGTTCCGT_L001_R1.fastq.gz
92f5f3ee337e4c8c5348e28c9a2a45bc *7_HNJ2JDRXY_GTTCTCGT-TGTTCCGT_L001_R2.fastq.gz
c83e4acaedd502cd555aad317b0db449 *8_HNJ2JDRXY_TCAGGCTT-ATCATGCG_L001_R1.fastq.gz
8bce9bfc2f5ba80f1be022aed2fff186 *8_HNJ2JDRXY_TCAGGCTT-ATCATGCG_L001_R2.fastq.gz
e4bc04e0cc7e51449121a2ddc9814011 *9_HNJ2JDRXY_CCTTGTAG-CCTTGGAA_L001_R1.fastq.gz
4d8a1338f2d788770a796eb494727865 *9_HNJ2JDRXY_CCTTGTAG-CCTTGGAA_L001_R2.fastq.gz
7621523966f9aac64fb97b2f77d347c4 *10_HNJ2JDRXY_GAACATCG-TCGACAAG_L001_R1.fastq.gz
d3c30f15e8bc24bfbb47f0d815473e1a *10_HNJ2JDRXY_GAACATCG-TCGACAAG_L001_R2.fastq.gz
5da95ec40eeb7693a4a35c6b387eca1c *11_HNJ2JDRXY_TAACCGGT-ATCGTCTC_L001_R1.fastq.gz
dc012e49a2f0a175988075ae92518a6b *11_HNJ2JDRXY_TAACCGGT-ATCGTCTC_L001_R2.fastq.gz
f0ca2845ee2f638e4686dd19f7b9afe0 *12_HNJ2JDRXY_AACCGTTC-CTAGCTCA_L001_R1.fastq.gz
f6f9e71896403b6cb6d5e10ade5fa274 *12_HNJ2JDRXY_AACCGTTC-CTAGCTCA_L001_R2.fastq.gz

Genome browser session
(e.g. UCSC)

not applicable

Methodology

Replicates

2 biological replicates were performed

Sequencing depth

150bp Paired End - Flowcell ID: 22J2HYLT
Lane Sample Name Paired End Data Yield(bp)
2 BP1 131,989,158 39.86 Gb
BP2 100,641,403 30.39 Gb
BP3 142,657,000 43.08 Gb
BP4 133,136,892 40.21 Gb
BP5 124,745,290 37.67 Gb
BP6 130,251,835 39.34 Gb
BP7 149,646,749 45.19 Gb
BP8 123,820,556 37.39 Gb
BP9 127,470,482 38.50 Gb
Total 1,164,359,365 351.64Gb

Antibodies

Anti-RNA polymerase II (8WG16) Santa Cruz Biotechnology sc-56767 (RRID: AB_785522)

Peak calling parameters

Peak calling was performed using MACS2 (v2.2.8). Peaks near transcription start sites (TSS) were annotated using bedtools closest (v2.30.0). The bamCoverage tool from deepTools (v3.5.1) was used to generate a normalized coverage track in BigWig format. Coverage was normalized using Reads Per Genomic Content (RPGC).

Data quality

Information has been described in the manuscript

Software

The enrichment pattern of around TSS is visualized using plot heatmap utility provided by deepTools.
